# Beyond Blind Trust in Hospital Data Warehouses: Evaluating Real‐World Data Accountability

**DOI:** 10.1002/lrh2.70118

**Published:** 2026-09-06

**Authors:** Akemi Morohashi, Satoshi Yamashita, Takahiro Imaizumi, Takuto Sano, Yachiyo Kuwatsuka, Masahiko Ando, Yoshimune Shiratori

**Affiliations:** ^1^ Nagoya University Hospital, Tokai National Higher Education and Research System Nagoya Japan

**Keywords:** data quality, evaluability, real‐world evidence

## Abstract

**Introduction:**

The reliability of real‐world evidence (RWE) depends on data accuracy and secondary users' ability to evaluate how data are generated and transformed. Although recent frameworks emphasize provenance, transparency, and auditability, their practical assessment in routine hospital settings remains unclear. This study examines the practical evaluability of real‐world data (RWD) and explores operational boundaries limiting data quality assessment within learning health systems.

**Methods:**

An empirical case study was conducted at a tertiary‐care university hospital in Japan. Data extracted from electronic health records and a clinical data warehouse were compared using 15 quality indicators derived from the *Data Quality Management Guidebook* of the Digital Agency of Japan (based on ISO/IEC 25012). Evaluability was assessed using a two‐step framework comprising empirical verification and contextual explanation from the perspective of research support personnel operating under routine institutional access permissions.

**Results:**

Evaluability varied substantially across clinical domains. Diagnosis records were generally verifiable and explainable, whereas medication‐related domains contained observations that could be verified but not fully explained by secondary users. Indicators including currentness and efficiency faced additional operational constraints, revealing boundaries between observable data characteristics and available explanatory information.

**Conclusions:**

Practical RWD use depends not only on observable data characteristics but also on the accessibility of information needed to interpret them. Evaluability boundaries reflect operational constraints rather than poor data quality. Rendering these boundaries visible may support more transparent and accountable RWD governance and inform future improvements through system design and data governance practices.

## 1 Introduction

The use of real‐world data (RWD) to generate real‐world evidence (RWE) has expanded globally to support regulatory decision‐making, postmarketing surveillance, and clinical research [1, 2]. Indeed, regulatory authorities worldwide have increasingly used RWE derived from routine electronic health records (EHRs) to support decisions such as approving new therapeutics and extending clinical indications [3, 4]. Although large‐scale public data infrastructures have been developed in Japan [5], intra‐institutional hospital data warehouses (DWHs) remain important platforms for flexible and timely clinical research.

However, data stored in DWHs are not direct replicas of EHRs. During extraction, transformation, and loading (ETL) processes, clinical context, transformation logic, and provenance information may become partially obscured from secondary users [6]. Consequently, while RWD continues to yield valuable evidence across diverse clinical and regulatory settings, determining whether a particular dataset is fit for a specific purpose remains a practical challenge. Recent reporting and governance frameworks, including RECORD, CODE‐EHR, and regulatory guidance documents, increasingly emphasize transparency, data provenance, and auditability as key elements supporting confidence in RWE [7, 8, 9, 10, 11]. These initiatives encourage researchers to understand the origin, transformation, and limitations of the data used to generate evidence. Simultaneously, routine healthcare information systems are primarily designed to support clinical care and operational requirements rather than secondary research use. Consequently, information relevant to understanding data generation and transformation is often distributed across multiple stakeholders, including clinicians, system vendors, information technology departments, and data analysts.

For learning health systems (LHS) to function effectively, stakeholders must be able to understand not only the content of data but also the circumstances under which those data were generated and transformed [12]. In routine hospital environments, however, researchers and technical staff involved in data extraction may not always have access to sufficient contextual information, such as system specifications, metadata, validation records, or operational knowledge necessary to fully explain observed data characteristics [6, 13, 14]. Such limitations do not necessarily indicate deficiencies in the underlying data or systems. Rather, they reflect the practical realities of using operational healthcare data across organizational and professional boundaries. The challenge is therefore not whether relevant information exists, but whether sufficient information is accessible to support transparent evaluation by secondary users within their operational context. Consistent with principles described in DAMA‐DMBOK [13], metadata and data lineage are key components of informed data use. Building on this perspective, this study examines evaluability, defined as the extent to which available evidence enables secondary users to understand, verify, and justify data characteristics relevant to a specific use case. Evaluability is therefore distinct from intrinsic data quality; it concerns the practical ability of users to assess and explain observed data characteristics using information available within their operational environment.

Although quality management frameworks are well established in manufacturing [15, 16] and regulated clinical trials (ICH‐GCP) [17, 18], practical approaches for assessing the evaluability of routinely generated RWD remain relatively underdeveloped [14]. Regulatory authorities have increasingly emphasized data provenance and auditability as key components of trustworthy RWE [1, 2, 19, 20, 21, 22, 23]. Nevertheless, the extent to which frontline technical staff can practically assess these attributes within complex hospital ETL environments remains unclear.

Building on our previous studies [24, 25], this study does not attempt to determine whether a DWH contains “good” or “bad” data, nor does it seek to evaluate the intrinsic quality of a specific dataset. Rather, it examines the practical evaluability of commonly proposed data quality indicators under real‐world operational constraints. Using 15 quality indicators derived from ISO/IEC 25012 [26], we investigate which aspects of data quality can be empirically verified, which can be explained using available contextual information, and where operational boundaries to evaluation emerge for secondary users within a hospital‐based LHS. Accordingly, this study aims to provide a practical framework for understanding not only what can be evaluated but also what remains difficult to evaluate under routine operational conditions.

## 2 Questions of Interest

This study addresses the following research questions regarding the evaluability and operational constraints of RWD within a hospital‐based LHS:

*(empirical feasibility). To what extent can standardized data quality indicators be empirically verified by secondary users in an operational DWH under routine access permissions?*

*(explanatory accessibility). To what extent can observed discrepancies and data implementation behaviors (e.g., data transformation or rounding logic) be understood and explained using information accessible to secondary users?*

*(operational boundaries). Which data domains are most vulnerable to evaluability boundaries, where available information within the operational context is insufficient to explain or validate observed data behaviors?*

*(design implications). What types of information provision and system design considerations, including Quality by Design principles, may improve the evaluability of RWD for secondary research use?*

## 3 Methods

### 3.1 Study Design and Setting

This study employs an empirical case study design at a tertiary care university hospital in Japan with 1076 beds.

**FIGURE 1:**
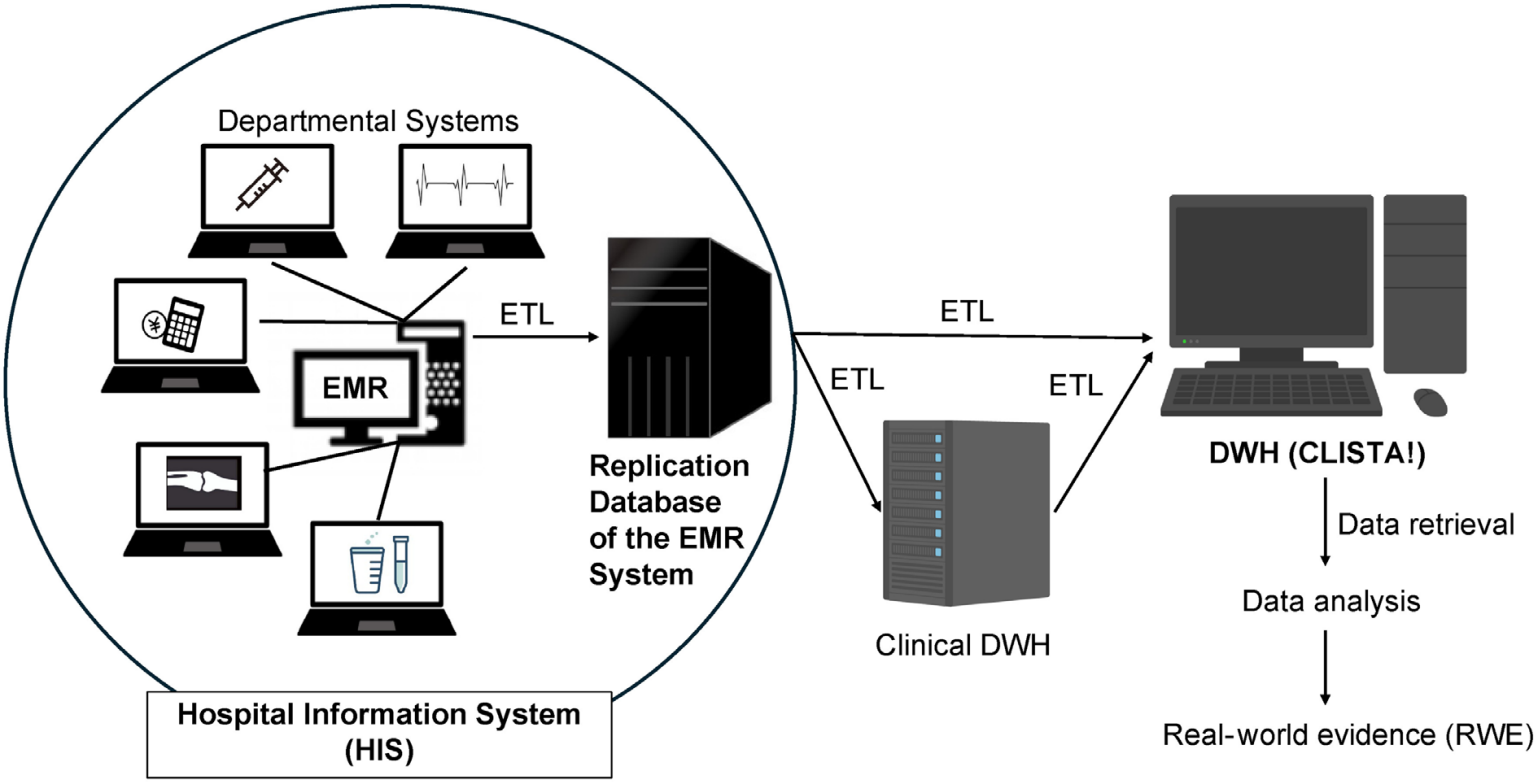
Conceptual overview of the HIS and workflow for RWD research.

The institution operates a Hospital Information System (HIS) integrating EHRs and departmental systems with a clinical DWH (CLISTA!, Medical Engineering Institute Inc.) that supports secondary use of clinical data for research and quality improvement activities (Figure 1).

The objective of this study was not to assess the intrinsic quality of a specific dataset. Rather, it was to evaluate the practical evaluability of commonly proposed data quality indicators when applied to operational hospital data under routine institutional conditions.

To reflect realistic research environments, all evaluations were conducted from the perspective of secondary users involved in data extraction and preparation (e.g., data managers and analysts). These users operated under routine access permissions and did not possess continuous administrative privileges or unrestricted access to system internals.

### 3.2 Data Sources and Study Domains

#### 3.2.1 Data Sources

Two data sources were used.

- *Reference source (HIS data)*: Reference data were obtained from a replication database synchronized with the operational EHR system. This database is routinely maintained for backup, auditing, and monitoring purposes and contains identical clinical content to the production environment. Because direct access to the live production database is restricted to ensure operational safety and system stability, the replication database served as the practical reference source for verification.
- *Target source (DWH data)*: Target data comprised records stored in the institutional DWH. These records are routinely generated through ETL processes that transfer information from the HIS into a format intended to support secondary use.

Hereafter, these datasets are referred to as “HIS data” and “DWH data,” respectively.

#### 3.2.2 Study Domains and Period

Three clinical domains were selected because they represent different levels of clinical and technical complexity:

1. Diagnosis records
2. Prescription orders
3. Injection administration records

The observation period extended from December 1, 2024, to January 31, 2025, and included approximately 460 000 records.

### 3.3 Evaluability Framework

**FIGURE 2:**
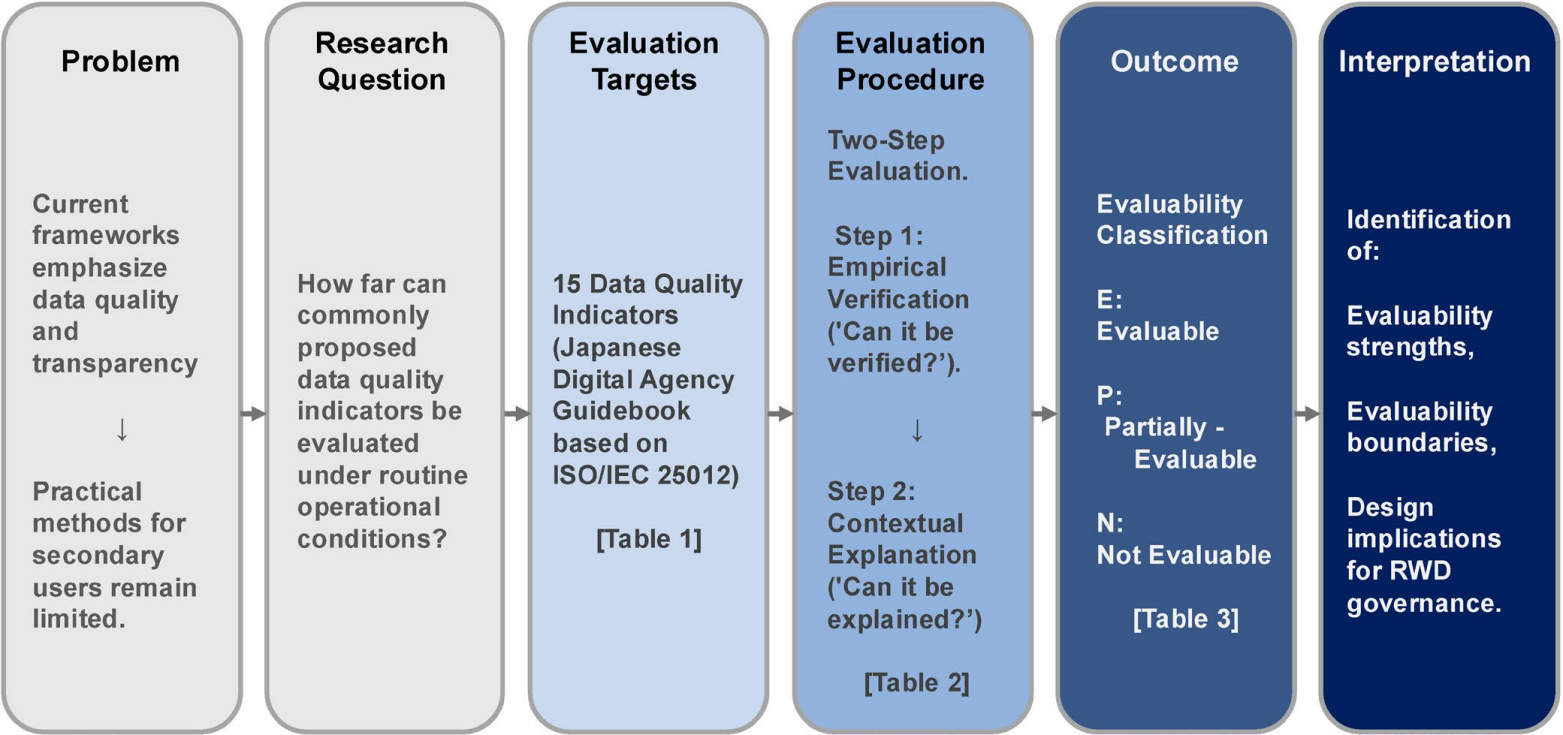
Conceptual framework for evaluating the evaluability of RWD.

The conceptual framework for assessing the evaluability of RWD is illustrated in Figure 2. The framework summarizes the evaluation targets, the two complementary evaluation perspectives, and the resulting evaluability classifications applied in this study.

Evaluability was defined as the extent to which secondary users could understand, verify, and explain observed data characteristics using information available within their operational environment.

Fifteen data quality indicators defined in the Data Quality Management Guidebook published by the Digital Agency of Japan [27], which is based on ISO/IEC 25012 [26], were adopted as evaluation targets (Table 1).

**TABLE 1:** Definition for the 15 data quality dimensions.

No	Dimension	Definition (the degree to which…)	Step 1: Empirical fact‐finding	Step 2: Contextual validation
1	Accuracy	Data values in the DWH correspond to those in the HIS, and for which any discrepancies can be objectively evaluated and explained based on accessible system information.	Direct record‐level comparison between HIS and DWH using common keys.	Validation of the legitimacy of discrepancies based on transmission and transformation logic specifications.
2	Completeness	All relevant records in the HIS are fully transferred to the DWH, and for which any missing or unique records can be systematically identified and justified.	Comparison of record counts and detection of orphaned records (data existing in only one system) using common keys.	Verification of whether identified orphaned records are objectively justified based on extraction, filtering, and de‐duplication specifications.
3	Consistency	Data values and coding structures remain logically consistent across systems and standards, and for which inconsistencies can be evaluated using available reference information.	Identification of logically inconsistent records through cross‐checking related fields and code mappings (e.g., Disease ID vs. ICD‐10).	Assessment of whether identified inconsistencies are explainable based on institutional rules, standard coding specs, or mapping logic.
4	Credibility	Data are perceived as trustworthy based on verifiable sources, documentation, and audit trails accessible to secondary users.	Identification of data sources and physical input pathways.	Confirmation of procedural integrity through technical evidence such as CSV (computer system validation) reports and authorization records.
5	Traceability	The origin, transformation, and modification history of data can be traced by secondary users using available documentation and system logs.	Observation of metadata (timestamps/user IDs) and availability of audit trails for record revisions.	Validation of change provenance based on audit‐log specifications and retention policies.
6	Currentness	Data in the DWH reflect recent updates in the HIS within an acceptable time frame, and for which the synchronization status can be verified under system constraints.	Perform direct reconciliation between HIS and DWH records to confirm update execution and identify latency.	Verification of documented evidence (e.g., system logs, schedules) to ensure the update mechanism is consistently performed as designed.
7	Accessibility	Authorized users can obtain necessary data and related information through established access mechanisms for research purposes.	Confirmation of data extraction feasibility within the scope of user permissions.	Validation of the appropriateness of access control policies and authorization procedures.
8	Compliance	Data structures and codes conform to external standards and institutional rules, and for which compliance mechanisms can be objectively verified.	Verification of the implementation status of standard medical codes.	Confirmation of conformance based on mapping rules and regulatory documentation.
9	Precision	Numerical data preserve their intended level of granularity and rounding rules, and for which precision loss can be objectively confirmed.	Comparison of numerical precision (e.g., decimal places, units) between HIS and DWH.	Verification of rounding and truncation rules based on data type definitions.
10	Confidentiality	Data are protected from unauthorized access through verifiable security and governance mechanisms.	Review of the implementation status of anonymization and access restriction measures.	Verification of compliance with institutional security policies and governance frameworks.
11	Efficiency	Data extraction is optimized through indexing and resource management, and for which processing performance can be objectively evaluated.	Verification of indexing status on major search keys and measurement of query response times.	Assessment of performance optimization policies and physical system constraints.
12	Understandability	Data structures, codes, and attributes can be correctly interpreted by users based on accessible metadata and documentation.	Review of the availability and clarity of metadata repositories and data dictionaries.	Validation of the definition's adequacy through technical documentation and user guidelines.
13	Availability	System operation time and service stability can be verified and justified through accessible operational records	Verification of declared system service hours (confirmation of officially defined operation hours via the DWH interface and user guidelines).	Verify the existence of institutional operational records that certify system stability and justify any observed service interruptions.
14	Portability	Data can be exported and reused across different platforms and analytical environments in standardized formats.	Assessment of supported export formats and verification of output consistency.	Confirming that output algorithms are certified to function according to system design specifications and interface documentation.
15	Recoverability	Data and system functions can be restored after failures based on documented backup and recovery procedures.	Physical verification of backup availability and execution logs.	Assess institutional recovery plans and validation records that certify the effectiveness of restoration procedures.

The indicators were selected because they represent a nationally recognized operational interpretation of internationally established data quality concepts and provide a practical framework for data quality management in Japan.

Rather than assessing these indicators solely as technical quality metrics, each indicator was examined from two complementary perspectives:

- *Empirical verification*: whether the indicator could be directly assessed through observable evidence obtained from HIS–DWH comparison.
- *Contextual explanation*: whether observed findings could be reasonably explained using information available to secondary users, including specifications, metadata, operational documents, and institutional knowledge.

### 3.4 Two‐Step Evaluation Procedure

**FIGURE 3:**
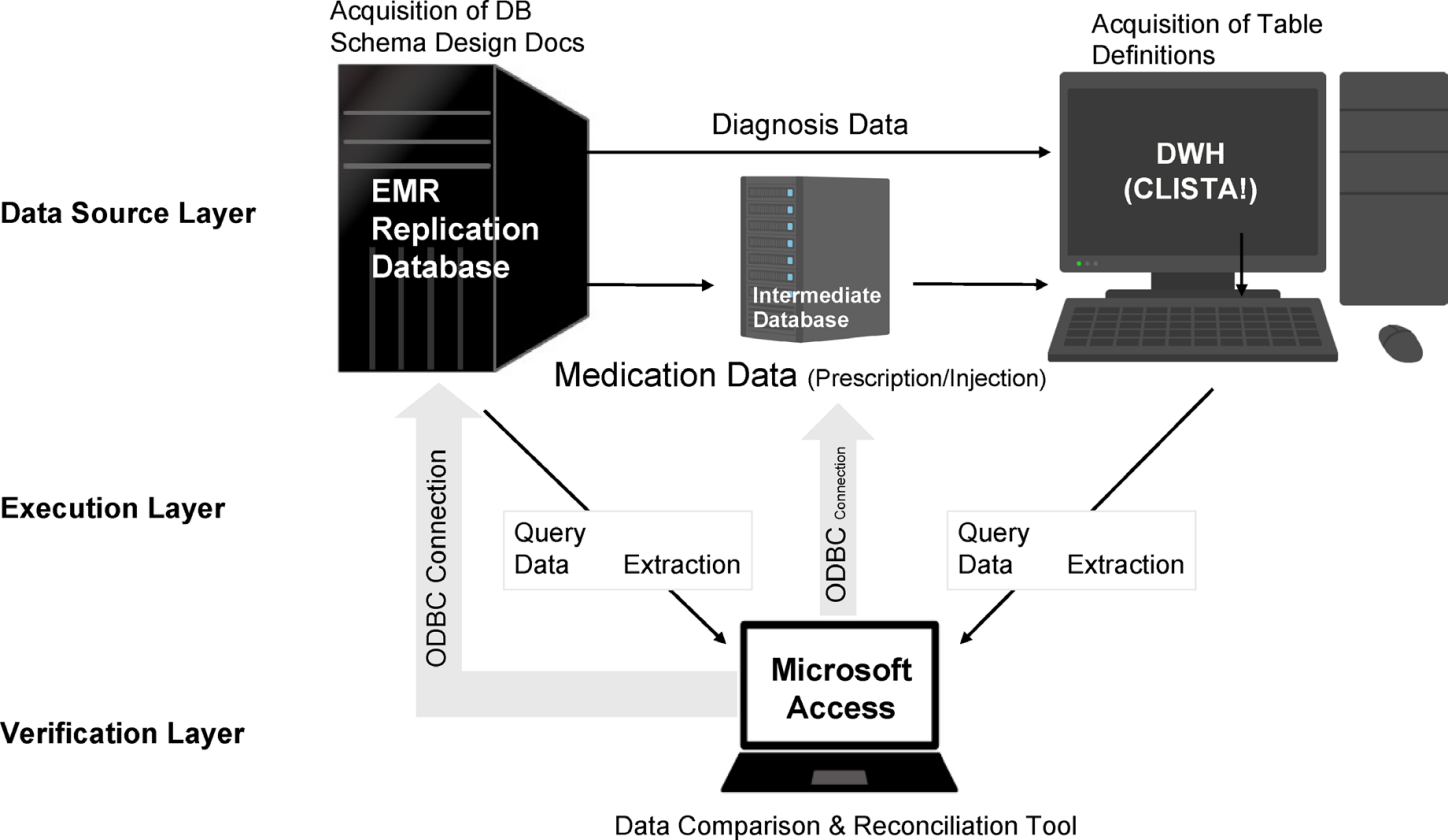
Systematic workflow for cross‐database validation between HIS and DWH.

A two‐step evaluation procedure was applied to all quality indicators (Figure 3).

The procedure was designed to evaluate the same observations from two complementary perspectives: empirical verification and contextual explanation.

Step 1: Empirical Verification (“What Was Observed?”)

Step 1 assessed whether data characteristics could be directly verified using observable evidence.

HIS and DWH records were compared using automated verification tools developed with SQL and Microsoft Access. Verification procedures included record‐count comparison, concordance assessment, missing‐value assessment, and consistency checks according to predefined evaluation rules (Table 2).

**TABLE 2:** Framework for data quality validation: dimensions, formulas, and specific methods.

Validation type	Dimension	Criterion (formula)	Matching rule/specific method	Evidence required for validation
Quantitative	Accuracy	Match rate (%) = *M*/(*M* + *N*) × 100 *M* (Matching): Number of records where values/existence match between the two datasets using Common Keys. *N* (Nonmatching): Number of records with discrepancies or orphaned data.	HIS vs. DWH: Extracted HIS data from the EMR replication database via ODBC using Microsoft Access. Data extraction (target): Extracted DWH data under identical criteria using system export functions (or different formats like CSV/Excel for Portability). Cross‐check: Conducted record‐level matching in Microsoft Access using Common Keys to verify the consistency of record counts and field values.	HIS‐to‐DWH data interface and ETL specifications
	Completeness	Match rate (%) = *M*/(*M* + *N*) × 100	HIS vs. DWH: Same as Accuracy. Verified record counts and identified orphaned records using common keys.	
	Currentness	Calculated based on the same formula as accuracy and completeness. Assessment of whether the data reflects the HIS state from the previous day without delay.	HIS vs. DWH: Same as accuracy. Focused on the latest records to verify synchronization from the previous day without delay.	
	Precision	Match rate (%) = *M*/(*M* + *N*) × 100	HIS vs. DWH (Granularity): Utilized the same HIS/DWH record sets in MS Access. Verified if the data granularity (decimal places, digits) in DWH is identical to the original HIS input.	
	Consistency	Match rate (%) $\frac{N_{\text{valid}\_\text{standard}\_\text{pairs}}}{N_{\text{total}}}\times 100\left(\%\right)$ Identification of whether the code‐to‐text relationship is logically and medically justified.	Within HIS (via Master): Utilized the same HIS record set in MS access as accuracy/completeness (as the denominator). Linked two related fields within HIS using standard masters as a reference in MS Access. Verified semantic matching; records with codes not found in the master are counted as nonmatching (*N*).	Internal master management regulations Standard code assignment and storage specifications in the DB Standard masters (e.g., MEDIS)
	Compliance	Match rate (%) $\frac{N_{\text{filled}\_\text{standard}\_\text{codes}}}{N_{\text{total}}}\times 100\left(\%\right)$ Identification of whether Step 1 results are logically explained by system specifications.	Targeted all records containing two or more standard codes (e.g., Disease Management ID and ICD‐10 code) from the HIS dataset used in the accuracy and completeness tests. Used MS Access queries to identify and count records with null or blank values for these standard codes. Calculated the vacancy rate (null rate) to evaluate the implementation status of standardized coding.	Internal master management regulations Master update specifications Standard code assignment and storage specifications in the DB
	Portability	Same as accuracy and completeness, comparing two different export formats (e.g., CSV vs. Excel). $\frac{\text{Number of matching records}}{\text{Matching records}+\text{Nonmatching records}}\times 100$	DWH Output (CSV vs. Excel): Exported data in multiple formats (e.g., CSV, Excel) using the same search criteria via DWH functions. Imported these exported files into MS Access. Performed cross‐checks to verify the consistency of record counts and data values across different file formats.	Evidence verifying that the system operates according to specifications (e.g., successful data export)
Qualitative/mechanism verification	Credibility	Assessment of the existence and integrity of historical metadata.	User‐perspective review of table definitions and cross‐DB integration specs to verify if the data structure and flow are demonstrably consistent.	Information to prove that the system operates according to specifications, such as integration and transformation specs, provided as evidence to the user.
	Understandability	Assessment of metadata clarity and availability.	Semantic documentation review: Assessment of metadata clarity, availability of data dictionaries, and consistency of field‐level definitions.	Institutional data governance guidelines: Validation of metadata adequacy through official user guidelines and technical data dictionaries.
	Traceability	Assessment of the existence and integrity of historical metadata.	Change provenance check: Observation of metadata (e.g., last‐updated timestamps) and availability of audit trails for record revisions or deletions.	Audit‐log specifications: Governance reports or validated logging policies defining the depth and retention of change histories.
	Efficiency	Assessment of operational stability and service levels.	Empirical performance testing: Measurement of query response times for standard datasets and identification of indexing status on major search keys.	Performance Qualification (PQ) reports: Technical documentation or physical schema designs specifying performance optimization and infrastructure policies.
	Availability	Assessment of the adequacy of database design based on performance optimization policies and physical schemas.	Verification of declared service hours: Direct confirmation of officially defined operation hours and maintenance windows via the DWH interface and user guidelines.	System operational records: Internal documents such as uptime metrics and unplanned outage logs to substantiate actual operational stability.
Infrastructure and operational verification	Accessibility	Assessment of the alignment between physical access and organizational disclosure rules.	Review of index optimization status, system administration logs, and tracing of user application workflows to ensure functional accessibility. Verification of system functional availability (e.g., access controls, backup/restore features) through manual review and log inspections.	Internal facility regulations, system user application forms, and documentation proving the system operates per specifications. Evidence to prove system operations per specifications, specifically regarding backup/recovery procedures and security protocols.
	Confidentiality	Assessment of adherence to formal data standards and structural requirements.	Security control review: Assessment of accessible security settings, user role definitions, and data protection measures visible to secondary users.	Security audit reports: Internal security policies, authentication/authorization design documents, and validated security audit evidence.
	Recoverability	Assessment of the reliability of recovery evidence.	Recovery procedure review: Observation of backup‐related metadata or status indicators available within the system interface.	Disaster recovery (DR) plans: Official BCP documents, backup execution logs, and validated restoration testing records.

Step 2: Contextual Explanation (“Why Was it Observed?”)

Step 2 assessed whether observations identified in Step 1 could be adequately explained using information available within the operational environment.

Available information sources included database specifications, ETL mapping documents, system manuals, operational procedures, and other institutional records.

When observed phenomena could not be adequately explained because relevant information was unavailable, inaccessible, incomplete, or distributed across stakeholders beyond the evaluator's scope, the observation was considered to have reached an evaluability boundary.

Such situations were interpreted as limitations in the accessibility of explanatory information rather than evidence of poor data quality.

### 3.5 Classification of Evaluability

For each quality indicator, evaluability was classified into three levels (Table 3).

**TABLE 3:** Observational scope and limitations in data quality verification across clinical categories.

No	Dimension	Step	Evaluation results					Barrier to full validation
			Findings/examples	Diagnosis	Prescription order	Injection administration	Overall	
1	Accuracy	S1	Diagnosis: 100% match (all 27 fields). Prescription/injection: discrepancies identified (3 and 11 items, respectively), including dosage rounding at the second decimal place.	E	E	E	Partially restricted	Specific transformation rules for drug data were unspecified, requiring an inference‐based approach for data verification.
		S2	Diagnosis: Fully verified via mapping specs. Prescription/injection: Validation failed due to missing Intermediate DB specifications and undocumented transformation logic.	E	N	N		
2	Completeness	S1	Match rate Diagnosis: 100% (59 678 records). Prescription: 81.2% (276 948 records). Injection: 99.0% (233 640 records).	E	E	E	Partially restricted	Drug transformation rules were inferred due to unspecified matching specs.
		S2	Diagnosis: Successfully verified via interface specs. Prescription/injection: Validation failed due to the lack of extraction/transmission logs between the intermediate DB and DWH. Reasons for record loss could not be justified.	E	N	N		Lack of detailed HIS transmission specs precluded verification of data loss or filtering logic.
3	Consistency	S1	Moderate agreement was observed in code‐pair checks (e.g., diagnoses: 4528/7598; injections: 617/674)	E	E	E	Partially restricted	Lack of documentation on local master‐data governance limited the interpretation of discordance.
		S2	Some discordance could be inferred (e.g., in‐house codes), but institutional mapping rules were unavailable.	N	N	N		
4	Credibility	S1	Presence of an intermediate database was suggested, but its extraction/transfer specifications were unavailable.	E	P	P	Partially restricted	Black‐box intermediate processing and missing system assurance documentation (e.g., CSV reports).
		S2	No formal system assurance evidence or validation reports were accessible.	N	N	N		
5	Traceability	S1	Update timestamps were observable; however, detailed revision histories were unavailable.	P	P	P	Partially restricted	Limited audit trails and nonretention of deleted records hindered change provenance verification.
		S2	Audit‐log specifications and validation evidence were not accessible	N	N	N		
6	Currentness	S1	Data extraction using update dates was not feasible for large‐scale prescription records.	E	N	E	Partially restricted	Physical query constraints (indexing issues) and undocumented intermediate synchronization logic.
		S2	Intermediate‐to‐DWH transfer specifications and propagation rules were unavailable.	N	N	N		
7	Accessibility	S1	Accessibility was evaluated only under the evaluator's own account.	P	P	P	Partially restricted	Limited verification of institution‐wide accessibility
		S2	Role‐based access policies and governance documents were unavailable.	N	N	N		
8	Compliance	S1	ICD‐10 coverage was 99.9%; HOT codes were populated in 82.1% (prescriptions) and 99.1% (injections, 0.9% dummy).	E	E	E	Partially restricted	Unclear rationale for coding exceptions.
		S2	Master lookups explained some blanks, but institutional governance could not be validated.	P	P	P		
9	Precision	S1	Identified numerical rounding (e.g., 2nd decimal place), indicating a loss of granularity compared with HIS.	—	E	E	Partially restricted	Undocumented transformation logic in the intermediate layer prevented justification of data changes.
		S2	No technical documentation described the rationale for this transformation.	—	N	N		
10	Confidentiality	S1	Security control and access‐procedure documentation was unavailable.	N	N	N	Not evaluable	Restricted access to security‐related evidence.
		S2	No security validation reports were accessible.	N	N	N		
11	Efficiency	S1	Indexing and query‐optimization information was unavailable to the evaluator.	P	P	P	Partially restricted	Lack of technical performance documentation and performance qualification (PQ) reports.
		S2	No performance qualification reports or infrastructure designs were accessible.	N	N	N		
12	Understandability	S1	Partial semantic documentation was available; however, record‐state flags remained ambiguous.	P	P	P	Partially restricted	Incomplete metadata and unclear flag semantics without a validated data dictionary.
		S2	No formal data dictionary or institutional governance evidence was provided.	N	N	N		
13	Availability	S1	Declared service hours were confirmed via the DWH interface and user guidelines.	E	E	E	Partially restricted	Information asymmetry regarding operational stability due to restricted access to infrastructure logs.
		S2	Actual operational logs and uptime records were inaccessible.	N	N	N		
14	Portability	S1	Data export was feasible. Confirmed that data could be extracted in standard formats (e.g., CSV, Excel) for external use.	E	E	E	Partially restricted	Absence of formal portability assurance.
		S2	No validation reports supported export reliability.	N	N	N		
15	Recoverability	S1	Backup and restoration logs were inaccessible.	N	N	N	Not evaluable	Lack of recovery‐related operational evidence.
		S2	No recovery procedure documents were available.	N	N	N		

*Note:* E, P, and N indicate the degree of evaluability under the study conditions (E: evaluable, P: partially evaluable, N: not evaluable) and do not represent data quality itself. S1 indicates empirical observability and S2 indicates availability of explanatory documentation.

The classification summarizes the extent to which observed data characteristics could be both verified and explained within the study setting.

- *E (evaluable)*: Observed data characteristics could be both empirically verified and adequately explained using information available within the operational environment.
- *P (partially evaluable)*: Observed data characteristics could be empirically verified, but explanatory information was incomplete, inaccessible, or insufficient to fully account for the findings.
- *N (not evaluable)*: Observed data characteristics could not be adequately verified and/or explained because of operational, technical, or access‐related constraints.

This classification was intended to characterize limitations in evaluability rather than limitations in intrinsic data quality.

### 3.6 Ethics and Reporting Standards

#### 3.6.1 Ethics Statement

The study was conducted under an institutional research program aimed at building a data infrastructure for the National Collaboration of Core H to generate RWE. The overarching program and its associated data quality assessments (DQAs) were reviewed and approved by the institutional ethics review committee of Nagoya University Hospital (Approval No. 2020‐0511). This study was conducted within the formally approved scope of this framework and in accordance with the ethical standards of the institutional review board and the Declaration of Helsinki (1975, revised in 2008).

#### 3.6.2 Consent

This study was conducted as a component of system infrastructure development intended to facilitate the secondary use of clinical data, rather than as a study involving secondary data use itself. Although real‐world clinical data were accessed during the validation process, data processing was performed strictly within the institutional environment, and no data were transferred to external entities. The institutional ethics review committee determined that individual informed consent was not required for this activity, as it posed minimal risk and focused on system verification and infrastructure development. This determination was formally approved in accordance with institutional and national ethical guidelines.

#### 3.6.3 Data Access and Security

Access to the EHR system and the institutional DWH was granted through a formal institutional authorization process. All analyses were conducted within a secured HIS network under controlled access conditions.

#### 3.6.4 Privacy Protection

Authorized research staff accessed identifiable clinical data solely within the secured institutional environment for verification and quality assessment purposes. In accordance with institutional privacy and security policies, all data handling and analyses were conducted within the protected HIS network, and no identifiable data were exported outside the institutional environment. These measures were implemented to prevent unauthorized access, disclosure, or secondary use of patient information.

#### 3.6.5 Data Integrity

Individual‐level data were not exported outside the institutional environment. All procedures complied with institutional security policies and national ethical guidelines regarding the secondary use of medical data.

## 4 Results

### 4.1 Categorization of Evaluability

Evaluability varied according to the extent to which observed data characteristics could be verified and explained using information available within the operational environment. Based on the combined outcomes of empirical verification (Step 1) and contextual explanation (Step 2), the 15 quality indicators were classified into three groups (Table 3).

The classification did not represent differences in intrinsic data quality. Rather, it reflected differences in the extent to which secondary users could evaluate and explain observed data characteristics within the scope of information accessible during routine operational activities.

### 4.2 Group 1: Indicators With High Evaluability

Diagnosis‐related indicators represented a domain in which both empirical verification and contextual explanation were consistently achievable.

Record‐level comparisons between HIS and DWH data demonstrated high concordance, and observed differences could be reconciled using information available through routine operational documents and system specifications. Consequently, secondary users were able to not only verify observed data characteristics but also explain them within the institutional context.

These findings indicate a high degree of evaluability for diagnosis‐related data. In this domain, the information available to secondary users was generally sufficient to support both empirical assessment and contextual understanding of the observed data characteristics.

### 4.3 Group 2: Indicators Reaching the Evaluability Boundary

Medication‐related domains, including prescription orders and injection administration records, exhibited a distinct evaluability boundary.

Several data characteristics identified during empirical verification could be directly observed through comparison between HIS and DWH records. Examples included differences in numerical values, rounding behaviors, and implementation‐specific processing patterns. However, in some cases, the information available to secondary users was insufficient to fully explain how these observations arose.

Importantly, these findings do not necessarily indicate errors, deficiencies, or inappropriate data processing. Rather, they illustrate situations in which observed data characteristics could be verified but could not be fully explained using the information accessible within the operational environment.

The gap between observation and explanation was particularly evident in domains involving complex transformation processes and specialized operational workflows. These results suggest that evaluability may vary according to the extent to which explanatory information is accessible to secondary users.

### 4.4 Group 3: Indicators With Limited Evaluability

A third group comprised indicators for which evaluation was substantially constrained by the availability of information required for assessment.

Indicators such as efficiency and currentness required information related to system performance, indexing structures, update histories, or other operational metadata that were not routinely accessible within the scope of this study. Consequently, neither empirical verification nor contextual explanation could be completed to the same extent as for other indicators.

These findings should not be interpreted as evidence of poor data quality. Rather, they reflect practical limitations in the evaluability of certain indicators when assessed from the perspective of secondary users operating under routine institutional access conditions.

### 4.5 Summary of Evaluability Across Clinical Domains

Across the three clinical domains, evaluability was influenced not only by observable data characteristics but also by the accessibility of information required to explain those characteristics.

Diagnosis‐related data generally demonstrated high evaluability, whereas medication‐related domains more frequently reached evaluability boundaries where empirical observations could not be fully explained using available information. Indicators requiring system‐level operational metadata showed the lowest evaluability because the necessary information was outside the scope routinely available to secondary users.

Overall, the findings suggest that the practical evaluation of RWD depends not only on the data themselves but also on the extent to which relevant explanatory information can be accessed and interpreted within the operational environment. This distinction became visible through the two‐step evaluation framework and provides a basis for identifying areas where additional transparency, metadata availability, or system documentation may improve the evaluability of RWD for secondary use.

## 5 Discussion

### 5.1 Evaluability as a Complementary Perspective to Traditional DQA

This study examines the practical evaluability of commonly proposed data quality indicators from the perspective of secondary users operating within a routine hospital environment. The findings suggest that challenges in evaluating RWD arise from not only the data themselves but also differences in the accessibility of information required to interpret and explain observed data characteristics.

Traditional DQA frameworks, including those proposed by Kahn et al. [14], focus on dimensions such as completeness, conformance, plausibility, and consistency. These frameworks provide essential approaches for assessing observable characteristics of data. Our findings do not challenge the value of these approaches. Rather, they highlight an additional perspective: whether secondary users can access sufficient information to understand and explain the results of such assessments within their operational context.

The concept of an evaluability boundary emerged when observed data characteristics could be empirically verified but could not be fully explained using information available to the evaluator. Importantly, reaching such a boundary does not necessarily indicate poor data quality, inappropriate system behavior, or deficiencies in governance. Instead, it reflects practical limitations encountered when data generated for clinical and operational purposes are reused across organizational and professional boundaries.

From this perspective, evaluability may serve as a complementary dimension to conventional DQA frameworks by helping organizations identify where additional contextual information, metadata, or explanatory resources may be beneficial for secondary data use.

Furthermore, limitations in evaluability within a single data source may be partially mitigated through triangulation across multiple data sources and consistency assessments, an approach increasingly used in contemporary RWE research [28].

### 5.2 Enhancing Evaluability Through Quality by Design (QbD)

Recent reporting and governance frameworks, including CODE‐EHR and regulatory guidance documents, increasingly emphasize transparency, traceability, and data provenance as key components of trustworthy RWE [1, 2, 19, 20, 21, 22, 23, 29]. Our findings suggest that achieving these goals depends not only on reporting practices but also on the availability of information generated and maintained throughout the data lifecycle.

In this study, some observations identified during empirical verification could not be fully explained using information routinely available to secondary users. Examples included transformation behaviors, operational processing rules, and system‐level metadata that were outside the scope of accessible documentation. Such situations do not imply that the information does not exist. Rather, the information may be distributed across multiple stakeholders, embedded within operational processes, or maintained outside the scope routinely accessible to researchers and data analysts.

These findings suggest that QbD principles may contribute to improving evaluability when incorporated into HIS and DWH infrastructures. In a QbD‐oriented environment, metadata related to data creation, modification, transformation, and management can be generated and maintained more systematically as part of routine operations. Such infrastructure‐level support may facilitate transparency, improve traceability, and enhance the ability of secondary users to understand and explain observed data characteristics.

From this perspective, transparency, provenance, and evaluability should be regarded as interrelated properties that are supported not only by documentation but also by the design and governance of information systems.

### 5.3 Implications for Governance in LHSs

LHSs rely on the continuous generation and reuse of data to support improvement in both research and clinical practice [12]. For such learning cycles to function effectively, stakeholders must be able to understand not only the content of data but also the circumstances under which those data were generated and transformed.

The framework proposed in this study provides a practical approach for identifying where evaluability is readily achievable and where additional contextual information may be required. Rather than assessing trust as an abstract concept, the framework enables organizations to examine the extent to which observed data characteristics can be independently verified and reasonably explained within their operational environment.

The implications of evaluability may be, particularly, relevant in research areas requiring detailed understanding of data generation processes, such as pediatric dosing studies, pharmacovigilance, and medication safety research. In such settings, transformation rules, calculation methods, or operational workflows that have minimal impact in routine care may become increasingly important when interpreting research findings.

Accordingly, improving evaluability is not solely a technical challenge. It requires collaboration among clinicians, information technology departments, system vendors, data managers, and governance bodies. By making explanatory information more accessible and systematically maintained, healthcare organizations may strengthen confidence in secondary data use while supporting sustainable learning and continuous improvement.

### 5.4 Limitations and Future Directions

Several limitations should be considered when interpreting these findings.

First, this study was conducted at a single tertiary‐care university hospital using one institutional HIS‐DWH environment. Evaluability may vary across healthcare organizations, system architectures, governance structures, and operational practices.

Second, the study intentionally adopted the perspective of secondary users operating under routine institutional permissions. Consequently, findings regarding evaluability boundaries may differ from those obtained by system administrators, vendors, or other stakeholders with broader access to technical information.

Third, the framework evaluates the practical ability to verify and explain data characteristics rather than intrinsic data quality itself. Therefore, the results should not be interpreted as direct evidence regarding the overall quality of a specific dataset.

Future studies should apply the framework across diverse healthcare institutions, data platforms, and clinical domains to assess its generalizability. Additional research is also needed to explore how evaluability relates to regulatory expectations for different use cases, including observational research, post‐marketing surveillance, and regulatory decision‐making.

## 6 Conclusion

This study proposes and applies a practical framework for evaluating the evaluability of routinely generated RWD within a hospital‐based LHS.

Using 15 quality indicators derived from ISO/IEC 25012 and a two‐step evaluation procedure, we examined not only whether data characteristics could be empirically verified but also whether those observations could be explained using information available to secondary users operating under routine institutional conditions.

The findings indicate that the practical use of RWD depends not only on observable data characteristics but also on the accessibility of contextual information required to interpret and explain those characteristics. Across several domains, empirical verification was achievable, whereas contextual explanation was limited by the availability of relevant information. These situations were conceptualized as evaluability boundaries.

Importantly, evaluability boundaries should not be interpreted as evidence of poor data quality or inadequate system performance. Rather, they reflect the practical realities of reusing operational healthcare data across organizational and professional boundaries.

The proposed framework offers a complementary perspective to traditional DQA by helping organizations identify what aspects of data can be evaluated, what can be explained, and where additional transparency or contextual information may improve secondary data use. By making these boundaries visible, the framework may contribute to more transparent, accountable, and sustainable use of RWD within LHSs.

## Funding

This work was supported by the Ministry of Health, Labour and Welfare and Japan Society for the Promotion of Science (JP 21K12750).

## Conflicts of Interest

The authors declare no conflicts of interest.

## Data Availability

The data used in this study are not publicly available due to legal and institutional restrictions regarding the protection of personal health information. Under the policies of Nagoya University Hospital, the clinical data cannot be shared or transferred to third parties to ensure patient privacy and data integrity.
